# Characterizing the literature on validity and assessment in medical education: a bibliometric study

**DOI:** 10.1007/s40037-018-0433-x

**Published:** 2018-05-23

**Authors:** Meredith Young, Christina St-Onge, Jing Xiao, Elise Vachon Lachiver, Nazi Torabi

**Affiliations:** 1 Department of Medicine, McGill University, Montreal, Canada; 2 Centre for Medical Education, McGill University, Montreal, Canada; 3 Department of Medicine, Université de Sherbrooke, Sherbrooke, Canada; 4 Health Profession Education Center, Université de Sherbrooke, Sherbrooke, Canada; 5 Library for Health Sciences, McGill University, Montreal, Canada

**Keywords:** Assessment, Validity, Bibliometrics, Medical education

## Abstract

**Introduction:**

Assessment in Medical Education fills many roles and is under constant scrutiny. Assessments must be of good quality, and supported by validity evidence. Given the high-stakes consequences of assessment, and the many audiences within medical education (e. g., training level, specialty-specific), we set out to document the breadth, scope, and characteristics of the literature reporting on validation of assessments within medical education.

**Method:**

Searches in Medline (Ovid), Web of Science, ERIC, EMBASE (Ovid), and PsycINFO (Ovid) identified articles reporting on assessment of learners in medical education published since 1999. Included articles were coded for geographic origin, journal, journal category, targeted assessment, and authors. A map of collaborations between prolific authors was generated.

**Results:**

A total of 2,863 articles were included. The majority of articles were from the United States, with Canada producing the most articles per medical school. Most articles were published in journals with medical categorizations (73.1% of articles), but Medical Education was the most represented journal (7.4% of articles). Articles reported on a variety of assessment tools and approaches, and 89 prolific authors were identified, with a total of 228 collaborative links.

**Discussion:**

Literature reporting on validation of assessments in medical education is heterogeneous. Literature is produced by a broad array of authors and collaborative networks, reported to a broad audience, and is primarily generated in North American and European contexts. Our findings speak to the heterogeneity of the medical education literature on assessment validation, and suggest that this heterogeneity may stem, at least in part, from differences in constructs measured, assessment purposes, or conceptualizations of validity.

**Electronic supplementary material:**

The online version of this article (10.1007/s40037-018-0433-x) contains supplementary material, which is available to authorized users.

## What this paper adds

Assessments must be of high quality, and validity is a key marker of quality. Recent reviews report suboptimal application of modern validity frameworks. Conducting a bibliometric study, we found that work is created by many authors, published across education and clinical journals, reports a variety of assessment approaches, and prolific authors work in highly interconnected networks. This suggests literature reflects a variety of perspectives intended for a range of audiences. The variability in validation practices identified in previously published systematic reviews may reflect disciplinary differences, or perhaps even differences in understanding of validity and validation, rather than poor uptake of modern frameworks.

## Introduction

Assessment is omnipresent throughout the continuum of medical education whether for admissions, to monitor knowledge acquisition, to follow trajectories of learning, to support maintenance of competence, or for formal gatekeeping for professional practice [1–7].  As such, it spans training levels, contexts, areas of medicine, and assessment modalities including multiple choice questions and high fidelity simulation contexts [8–12]. Regardless of the multiple contexts and purposes, it is imperative that the assessments put in place in medical education are of the highest quality.

One of the markers of quality assessment is the validity evidence available to support the use and the interpretation of the assessment scores. There have been recent literature reviews reporting that published validation studies are falling short of recommended practices [13–16]. Cook et al. [14, 15] have suggested that we need to engage in better knowledge translation of validation frameworks to ensure increased quality in the assessment literature. Knowledge translation efforts to document the gaps between recommendations and actual validation practice implicitly assume that there is one ‘best’ framework that should be translated (e. g. [16–19]). However, medical education has been labelled as an emerging field, informed by various disciplines [20, 21], and intended for several different audiences, whether clinical, specialty-specific, educational, or focused on communicating to researchers in the field [20]. It is therefore possible that the medical education literature may facilitate the coexistence of different frameworks, or even conceptualizations of validity. St-Onge et al. have recently documented at least three different conceptualizations of validity present in the health professions education (HPE) literature [22], and the observed variation in validation practices [14, 15] could also be reflecting the differences across the disciplines that inform HPE. More specifically, some documented differences in validation practices reflect various validity frameworks or theories, from notions of the ‘trinity’ (construct, content, and criterion) [23], the unified theory of validity [24], and an argument-based approach to validation [25]. If a lag in knowledge translation [14, 15] and differences in conceptualizations of validity can result in differences in observed differences in validation, could there be other potential factors that could explain the observed variation in validation practices?

It may be possible that an analysis of the breadth, scope, and characteristics of the assessment literature in medical education may provide a window into understanding the previously reported gap between actual and recommended practices [15]. We employed bibliometric approaches [26–28] to document the breadth, scope, nature, and heterogeneity of the literature reporting on validation of assessments within medical education literature. Bibliometric analyses can provide means to describe a body of published work, which is based, in part, on the assumption that published literature reflects the state of knowledge in a given field [26–28]. These analyses can provide data concerning publication characteristics, patterns, and estimates of absolute and relative productivity [21, 26, 29, 30]. Several bibliometric studies have been published in medical and health professions education [26, 29–32], but many of these have limited scope by limiting to one journal, or one type of journal, in order to characterize medical education as a field [29, 30]. Rather than focusing on medical education as a field, or publication patterns of a given journal, here we focus on describing the literature on validation of assessment in medical education across multiple publication contexts. In this study, we identify articles reporting on validity of assessment within the broad field of medical education in order to explore the scope and variability of work on validation of assessments.

## Method

For the purpose of this bibliometric study, individual publications reporting on validity in the context of learner assessment in medical education are the units of analysis, as the data regarding the publication (such as where it is published, by whom, and when) are of interest, rather than the content of the article itself.

### Identification of relevant articles

In order to locate articles that reported on validation within the medical education literature on assessment, we searched the following electronic databases: Medline (Ovid), Web of Science, ERIC, EMBASE (Ovid), and PsycINFO (Ovid). The scope of the search was focused on the concepts “Measurement”, “Validity”, and “Medical Educational Area” (including postgraduate medical education and undergraduate medical education). See Appendix 1 of the online Electronic Supplementary Material for the full Medline search strategy. The search was limited to French and English articles published between 1999 and May 2014, as 1999 marked the release of a revised validation framework from the Standards for Educational and Psychological Testing [33] and the search was executed in May of 2014. All identified studies from the search strategies were imported to RefWorks (*n* = 20,403) and exact duplicated records were removed. After manual screening of titles and abstracts, 17,170 additional articles were excluded as they were duplicates or they did not meet inclusion criteria.

### Inclusion and exclusion criteria

Following the removal of duplicates, two independent reviewers (ML, EVL) screened titles and abstracts for inclusion criteria, with disagreement resulting in adjudication by a third (CSTO) or fourth team member (MY). Additionally, a third reviewer (CSTO) coded the first 200 titles and abstracts to further refine inclusion and exclusion criteria. Finalized inclusion and exclusion criteria were applied to the entire database (including those included in the first iterative step), and 10% of all articles were triple reviewed to ensure consistency in the application of the inclusion and exclusion criteria. Inclusion criteria were that articles must: 1) refer to assessment of learners (rather than program evaluation); 2) refer to assessment of competence, performance, or skills; 3) include a medical learner (be they undergraduate, postgraduate, or physician being assessed in the context of continuing professional development; including admissions and selection); and 4) be primary research.

**Fig. 1:**
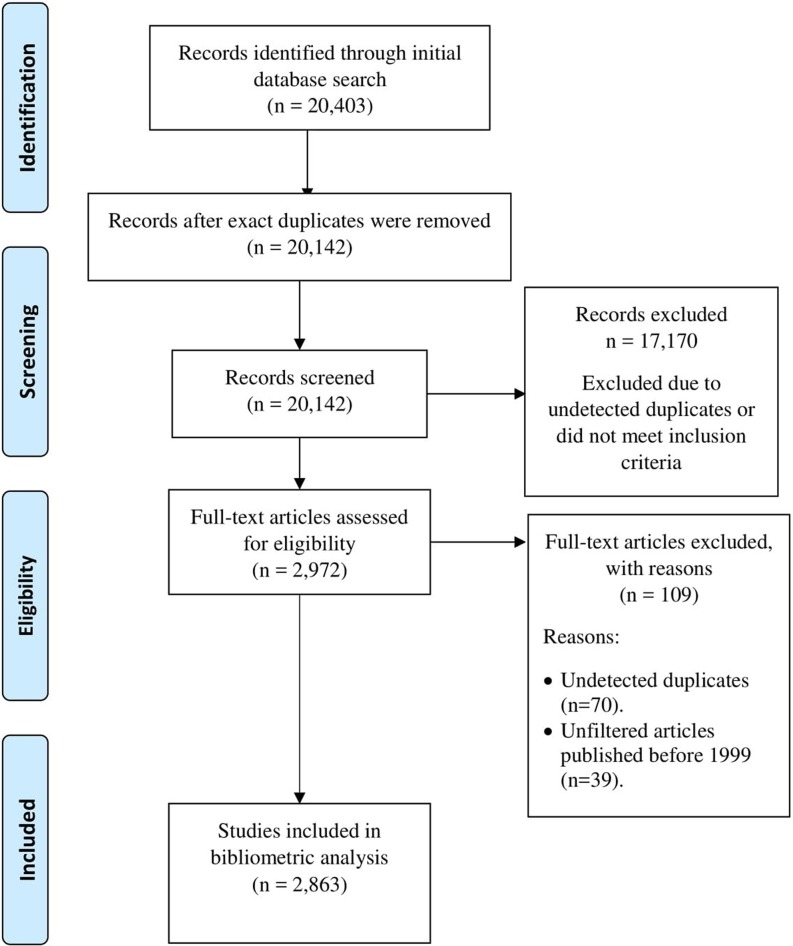
PRISMA (2009) flow diagram

Fig. 1 illustrates the PRISMA [34] flow diagram for the literature selection process. Abstract, title, authors, journal, publication year, and first author information of selected publications were exported to Microsoft Excel for descriptive analysis.

### Analysis

#### Interrater agreement

An initial calibration round was conducted where two reviewers (ML, EVL) applied the inclusion and exclusion criteria for 200 titles and abstracts. Following this, inclusion and exclusion criteria were refined and applied to three rounds of 500 titles and abstracts. Interrater agreement was calculated for each round, and recalculated for every 1,000 titles and abstracts. Interrater agreement was calculated by raw percent agreement between two coders (ML, EVL) for inclusion/exclusion.

#### Publication by year

A frequency count of papers published per year was performed to examine the historical pattern of publication for literature on validity of assessment in medical education.

#### Geographic representation

In order to explore the dispersion and relative productivity of literature reporting validity evidence for assessment in medical education, we recorded the reported country of the first author and conducted a frequency analysis. We calculated the ratio of number of publications per country represented in articles included in our study to the number of medical schools within a given country (as in Doja et al.) [29]. This ratio allows a more direct comparison of productivity across countries. The number of medical schools contained in each country was obtained from the World Directory of Medical Schools developed by the World Federation for Medical Education (WFME) and the Foundation for Advancement of International Medical Education and Research (FAIMER) [35]. We used the number of schools reported by WFME and FAIMER for 2016; we did not control for potential changes in number of medical schools across time in this analysis.

#### Journals and publication categories

Medical education is a multi-disciplinary field of study (e. g., medicine, education, cognitive psychology) that crosses multiple contexts (e. g., simulation, classroom, or workplace-based) [20]. Therefore, we examined the variability of journals by relying on the US National Library of Medicine (NLM) Broad Subject Terms for Indexed Journals, where each journal is categorized according to its primary content focus [36]. In order to mobilize these data, we searched the NLM database for the journals included in our study, and indicated which category/categories were assigned for each journal. The frequency of occurrence for each individual journal and each category of journal was tabulated for all articles included and subjected to descriptive analysis.

#### Scope of reported assessments

In order to capture assessment tools, techniques, topics, and approaches included in validation studies within medical education, we searched titles and abstracts for the following keywords (list generated collaboratively by the authors with knowledge of assessment and medical education): Clinical Encounter, Interview, Key Feature, MCQ, Mini-CEX, Multiple Mini Interview or MMI, OSAT, OSCE, Script Concordance Test or SCT, Short Answer Question or SAQ, Simulation, Simulator, Technical Skill Assessment, Test, and Exam.

#### Authors and author network

Sole, last, and first authors may be more likely to be considered influential members within the medical education community, and as such we focused this analysis of authors on sole, first, and last authors. For each sole, first, and last author, we documented the frequency with which they occurred in any of the papers included in the study and calculated the mean, standard deviation, and mode, of the number of publications for each of these authors.

Author networks (e. g. Doja et al.) [29] can be utilized to identify influential researchers and field of focus in medical education and collaborative work between researchers in medical education. To examine the authors of influence and their interrelationships, we set a cut-off of two standard deviations above the mean number of publications by an individual author in the database; this meant that to be considered an ‘author of influence’, one must have published a number of papers at least two standard deviations above the mean number of publications by a single author within our database. For each author of influence, a web search was conducted to identify their training background (restricted to MD or equivalent and PhD or equivalent), and specific area of training (for MD) or study (for PhD). An authorship network diagram was developed for these authors of influence by identifying the frequency of collaborations among authors of influence.

Visual inspection of the author network identified groups that were less well integrated into the entire author network. We identified the papers within our database published by these less-well-integrated groups and attempted to describe the commonalities across publications generated from these groups of authors.

## Results

### Interrater agreement

For initial calibration, interrater agreement was calculated following 200 title and abstract reviews (74% raw agreement between two raters). A team discussion resulted in refinement of the inclusion and exclusion criteria, followed by three more calibration rounds of 500 titles and abstracts (agreement ranged from 75 to 80% with disagreements adjudicated by CSTO or MY). Following these calibration rounds, interrater agreement was calculated for every 1,000 titles and abstracts reviewed to ensure consistency. Interrater agreement ranged from 88 to 93%, with disagreements adjudicated by CSTO or MY.

### Dataset of articles

Of an initial 20,142 citations, 2,863 publications between 1999 and May 2014 met the inclusion criteria and were included in the analysis (Fig. 1).

### Publication by year

The number of articles published per year ranged from 53 to 391, as shown in Fig. 1 of the online Electronic Supplementary Material, with an increase in the number of publications per year, particularly in the last decade.

### Geographic representation

A total of 69 unique countries across six continents were represented in our set of articles. Publications from North America accounted for 60% of all articles (*n* = 1,719), followed by Europe (25.6%, *n* = 734), Asia (9.1%, *n* = 259), Oceania (3.5%, *n* = 101), South America (0.9%, *n* = 27), and Africa (0.8%, *n* = 23). For countries with 10 or more publications, a ratio of number of publications to number of medical schools within that country was used to determine a rank of relative productivity. The United States led all countries with 1,366 publications followed by the United Kingdom with 340 publications; however, Canada ranked first and the Netherlands ranked second in relative productivity (Table 1).

**Table 1:** Total number of publications by country and relative productivity rank (ratio of number of publications by number of medical schools within that country)

Country	Number of articles	Percent of articles	Number of medical schools	Ratio (*n* of publications/*n* of schools)	Relative productivity rank
United States	1366	47.7	177	7.7	4
United Kingdom	340	11.9	53	6.4	5
Canada	335	11.7	17	19.7	1
Netherlands	128	4.5	10	12.8	2
Australia	81	2.8	20	4.1	7
Germany	48	1.7	41	1.2	14
France	36	1.3	53	0.7	15
Denmark	35	1.2	4	8.8	3
Pakistan	27	0.9	91	0.3	20
Ireland	27	0.9	7	3.9	9
China	26	0.9	184	0.1	23
Belgium	25	0.9	7	3.6	11
Japan	24	0.8	83	0.3	21
India	23	0.8	353	0.1	25
Switzerland	23	0.8	5	4.6	6
Taiwan	22	0.8	12	1.8	13
Iran	21	0.7	57	0.4	18
Saudi Arabia	20	0.7	31	0.7	16
New Zealand	20	0.7	5	4.0	8
Israel	19	0.7	5	3.8	10
Sweden	17	0.6	7	2.4	12
Brazil	17	0.6	209	0.1	24
Malaysia	14	0.5	26	0.5	17
Mexico	14	0.5	77	0.2	22
Spain	13	0.5	40	0.3	19

Countries with 10 or more publications are reported

### Journals and publication categories

#### Specific journals

The articles included in the study were published in 614 unique journals. The journals with 15 or more publications are listed in Table 1 of the online Electronic Supplementary Material. Articles included in our study were most commonly published in: Medical Education (number of articles = 214; percent of included articles = 7.4%), Academic Medicine (163; 5.7%), Medical Teacher (134; 4.7%), Journal of General Internal Medicine (88; 3.1%), Advances in Health Sciences Education (85; 3.0%), American Journal of Surgery (80; 2.8%), BioMed Central Medical Education (72; 2.5%), Academic Emergency Medicine (67; 2.3%), Teaching and Learning in Medicine (62; 2.1%), and Surgical Endoscopy (60; 2.1%).

#### Journal categories

Each unique journal was classified into category or field of study using the NLM Broad Subject Terms for Indexed Journals [36]. The 614 unique journals reflected 94 unique categories. Of all publications, 26.9% were associated with the category Education, 19.9% with the category General Surgery, and 9% of articles were found in journals with the category Medicine. To understand the nature of the journals in which articles in our database were found, we also analyzed the number of unique journals represented in each category. Meaning, we focused on the number of unique journals, in which articles in our database were published, in each category. When examining the number of unique journals, the category Medicine contained 15.8% of the unique journals (97 unique journals), the category Surgery included 12.7% (78 journals), and Education included 5.7% of journals (*n* = 35). Categories representing at least 1% of total publications can be found in Table 2 of the online Electronic Supplementary Material.

**Table 2:** Publications by keywords

Keywords	Number of articles	%
Simulation	432	15.1
Simulator	417	14.6
OSCE	249	8.7
Interview	216	7.5
MCQ	87	3.0
OSATs	83	2.9
Mini CEX	54	1.9
Script concordance Test	38	1.3
Multiple mini interview	30	1.1
Clinical encounter	29	1.0
Key feature	11	0.4
Short answer questions	10	0.4
Technical skill assessment	6	0.2
Exam	1,211	42.3
Test	1,398	48.8
Exam & test (Absolute^a^)	1,072	37.4

^a^Absolute indicates ‘Exam’ or ‘Test’ was present in the title or abstract without the presence of another other keywords

### Scope of reported assessments

Each keyword and the associated frequency within all publications are listed in Table 2. Simulation and simulators were the most common assessment context (20.7% of included papers), with OSCEs being the single most frequently reported assessment approach (8.7% of papers). OSATs (2.9%) and Mini-CEX (1.9%) were the most frequently reported assessment tools.

### Authors

Within the 2,863 publications, we identified 4,015 unique first, sole and last authors. Each of these individuals were named as an author for an average of 1.40 publications in the database (standard deviation = 1.38, range 1–35 publications, and mode of 1 publication per author). In the publications reviewed, we identified 97 unique sole authors, 2,172 unique first authors, and 2,040 unique last authors.

To identify ‘authors of influence’ for inclusion in the author network analysis, we identified all of those with more than five publications (mean + 2SDs = 4.16 publications, rounded up to be conservative), representing 2.2% of all authors in our database. Of the 4,015 unique authors, a total of 89 had five or more publications. Author degrees and fields of study or practice were highly variable, as can be seen in Table 3. See Appendix 2 in the online Electronic Supplementary Material for a list of authors with five or more publications (where they were first, last, or sole author) included in our analysis.

**Table 3:** Description of academic and clinical backgrounds of authors with five or more publications included in this study

Area of training	*n*	%
*MD*	46	51.7
– Surgery and surgical subspecialties (Otolaryngology-head and neck surgery, Oncology, Gynaecology, Urology, Orthopaedics)	27	30.3
– Medicine (Internal medicine, Anaesthesiology, Critical care)	13	14.6
– General practice (Family medicine, Paediatrics, Emergency medicine)	4	4.5
– Other (Psychiatry, Dermatology)	2	2.2
*MD/PhD (PhD area of study reported)*	16	18.0
– Education (Education, Educational psychology, Educational assessment, testing or measurement)	4	4.5
– Health Professions Education (Medical education, Clinical education)	7	7.9
– Psychology (Social psychology, Quantitative psychology)	2	2.2
– Medical sciences	3	3.4
*PhD*	27	30.3
– Education (Educational psychology, assessment, testing, and measurement)	9	10.1
– Health Professions Education	2	2.2
– Psychology (Cognitive psychology, Medical psychology, Psychometrics or quantitative psychology)	9	10.1
– Health studies (Public health, Human development)	3	3.4
– Sciences and medical sciences (Kinesiology, Nursing science, Nuclear physics)	4	4.5
*Total*	89	100.0

### Author network

**Fig. 2:**
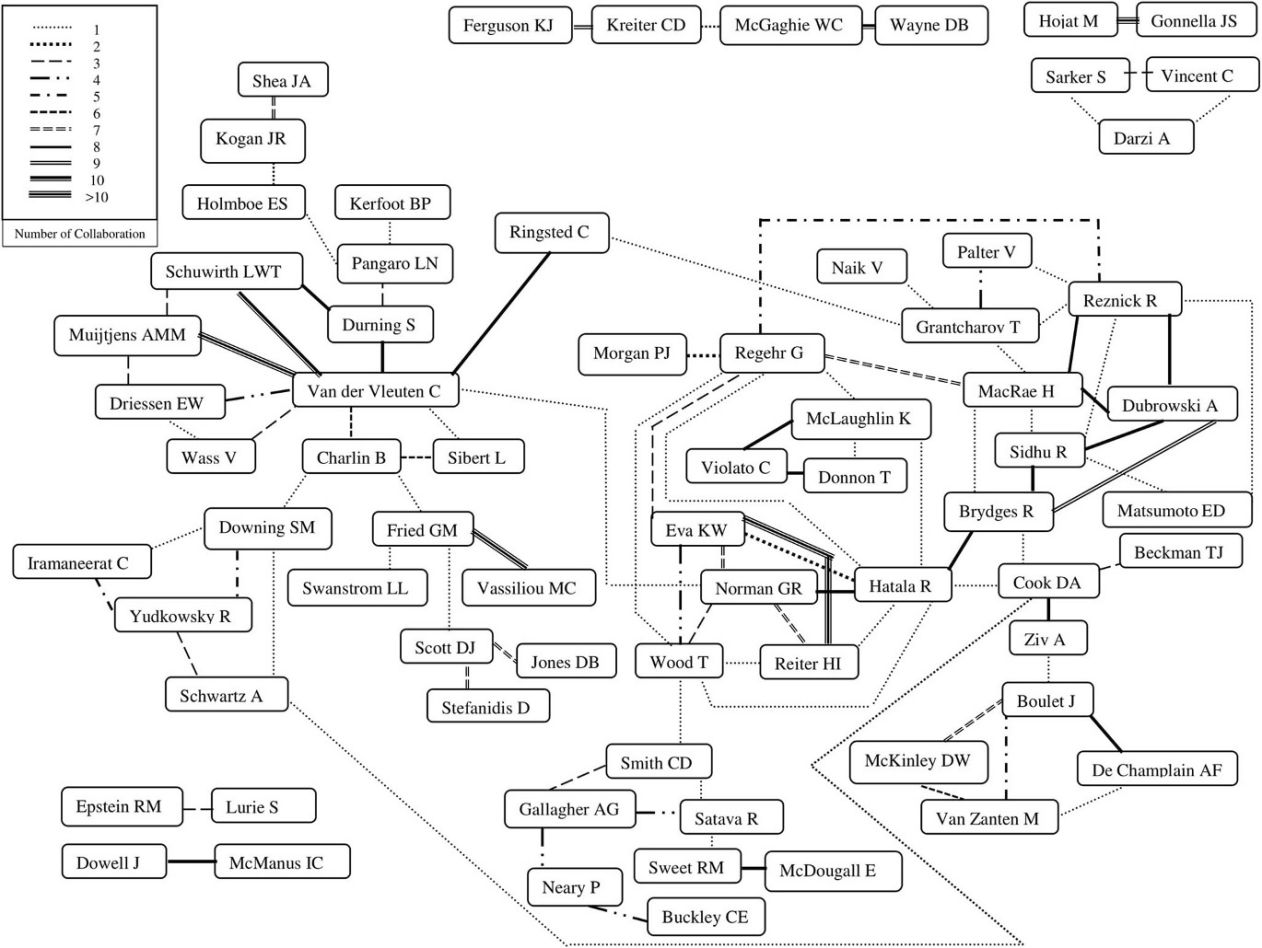
Author Collaboration Network of 89 identified unique authors with 5 or more publications included in our study

Amongst the 89 identified authors with five or more publications within our article set, a total of 228 different collaboration combinations were identified, indicating a highly-interconnected group of authors. Twenty percent (20.3%, *n* = 580) of articles in our database included one author of influence in the author list, 7% (6.9%, *n* = 198) included two authors of influence, nearly 1% (0.9%, *n* = 27) included three, and three articles in our database included four authors of influence. Repeated collaborations between authors are depicted in Fig. 2, likely indicating long-term successful collaborative relationships.

When examining the author network, it is clear that some prolific authors are strongly embedded within an interconnected network, while other authors are less centrally embedded in the author network. As discussed in Doja et al., a careful analysis of author networks, with consideration for relative productivity, can provide insight, or at least speculation, regarding what factors may be facilitating productive collaboration [29]. Our interpretation of the author network is based on the papers included in our study, and perhaps could be considered rather under-nuanced. However, we examined articles in our database produced by less-well-integrated teams of prolific authors in order to identify commonalities across publications, and speculate regarding the nature of their collaborations. Across less-well-integrated teams of prolific authors, we identified commonalities within teams in terms of collaboration around a common: construct, contexts, specialty-specific skill or task, tool, modality of assessment, assessment approach, goal or purpose of assessment, or approach to analysis. A summary can be seen in Table 4.

**Table 4:** Examples of commonalities across publications in less-well-integrated groups of prolific authors

*Group of authors*	*Assessment construct*
– Hoja and Gonnella	– Empathy
	*Context of assessment*
– Boulet, Van Zanten, DeChamplain, and McKinley	– High stakes assessment: licensure and certification
– Dowell and McManus	– High stakes assessment: undergraduate admissions
	*Assessment of a specialty-specific skill*
– Sarker, Vincent, and Darzi	– Laproscopic technical skills
– Fried, Vassiliou, Swanstrom, Scott, Jones, and Stephanidis	– Laproscopic technical skills: simulation contexts
	*Assessment tool*
– Kogan and Shea	– Mini-CEX
– Hojat and Gonnella	– Jefferson Scale
	*Assessment modality*
– Smith, Gallagher, Satava, Neary, Buckley, – Sweet, and McDougall	– Virtual reality simulator
	*Assessment approaches*
– McGaghie and Wayne	– Mastery learning
– Epstein and Lurie	– Peer assessment
	*Approaches to analysis*
– Ferguson and Kreiter	– Generalizability analysis

We can speculate regarding some clusters of authors within our network. Prolific authors who are less-well integrated in the network seem to cluster around the constructs measured, the assessment purpose or contexts, the specialty in which the assessment occurs, particular tools or approaches, and the choice of statistical analysis. Geographic location appears to be a facilitator for collaboration for some (e. g. highly integrated groups in Canada, United States, and the Netherlands).

## Discussion

This study reports a comprehensive bibliometric profile of the literature reporting on validation of assessment within medical education between 1999 and 2014. The amount of literature reporting on validation of assessment appears to be growing rapidly, with the majority of publications generated in North American contexts. The largest number of publications are produced in the United States, the United Kingdom, and Canada; however, the countries with the largest proportional productivity (number of papers per medical school) are Canada, the Netherlands, and Denmark—similar to findings reported by Doja et al. [29]. The largest number of articles were published in the journals Medical Education, Academic Medicine, Medical Teacher, and Journal of General Internal Medicine.

When attending to the categories of journals, relying on the NLM Broad Subject Terms for Indexed Journals [36], Education represented the category with the largest number of publications, meaning that the largest number of papers were published in journals classified in Education. However, General Surgery and Medicine represented the largest proportion of journals—meaning literature reporting on validation of assessments is spread broadly across a large number of journals in Medicine and Surgery, perhaps rendering some of the assessment literature difficult to synthesize.

The primary observation from this bibliometric profile is the heterogeneity present in the literature on assessment and validity in medical education—heterogeneity in authors, training backgrounds, location, journal of publication, publication domain, and assessment topic or tool. This heterogeneity has been noted in more restricted bibliometric studies (e. g. [26, 29, 30]) and has been expanded on here by including Medicine and Education journals in our search and analysis approach. Our findings align well with these earlier bibliometric studies, suggesting that the integrated nature of medical education as a field contributes to its breadth in terms of published literature. Furthermore, our study demonstrates the rapidity of growth of this literature base [13, 22, 29]. Here, we note a similar growth in the assessment literature with notable expansion since 1999. We can offer no current specific evidence-informed explanation for the growth pattern observed in this study; however, speculative considerations include expanded roles of assessment within medical education (e. g., assessment for learning, programs of assessment, etc.), and possible increases in available avenues for publication.

The literature included in this study represented a broad range of assessment tools and approaches, generated by a large group of authors (a total of 4,015 authors listed on a total of 2,863 papers). A group of 89 authors were identified as ‘authors of influence’, having published more than five papers included in our database, and appeared as authors on 1,069 of the articles in our study. These authors represented MDs, PhDs, and MD/PhDs from a variety of clinical specialties and disciplinary fields, and the majority represent a highly-integrated group of authors. Prolonged and highly-integrated patterns of co-authorship may suggest that the multiple perspectives and training backgrounds represented in these collaborations are contributing to the literature in a valuable and meaningful way.

Many prolific authors were broadly connected; however, other authors were less-well centrally embedded in the authors network. An examination of assessment approach, assessment tool, construct of interest, context of assessment, and discipline was not able to fully explain the presence or absence of collaborative links. Similar institutional affiliations appear to facilitate collaborative links (e. g. McGaghie and Wayne); however, several long-term collaborations appear to happen across institutions. While entirely speculative, we believe that some clusters of authors may conceptualize validity, or approach validation, very differently. It may not be unreasonable to assume that an approach to validation would be very different if focused on longitudinal programmatic assessment than if focused on ensuring the appropriateness of assessing within a simulated, or virtual reality, environment [25, 37]. We hypothesize that different conceptualizations of validity, or different approaches to validation, may be one factor that underlies the likelihood of collaborative links between individuals within our network. We have some indications that individuals who share similar understandings of a given construct are more likely to collaborate, or likely to engage with each other’s work; however, documenting this phenomenon remains in its infancy [38]. We also speculate that individuals with different conceptualizations of validity, or different approaches to validation, would be unlikely to collaborate extensively. This remains an area for future research, but approaches to validity and validation may be an important underlying factor in predicting successful collaborative relationship within assessment in medical education.

This study has limitations. The work reported here focuses solely on the meta-data available for literature on validity of assessment within medical education, and as such can speak to the breadth, scope, and nature of the published literature, but not to its specific content and quality. Examination of the context, content, and quality were beyond the scope of the current study, however represent important avenues for future research. Given the search strategies and databases used, this may have led to an overrepresentation of papers published in English language venues, or an overrepresentation of papers from North America. Another limitation of this study could be how we structured the author search in order to identify authors of influence. We decided to focus our analysis on first, last, and sole authors, and adopted the assumption that authors of influence would be more likely represented within those author positions. We recognize that this is an assumption, and the meaning of author order within health professions education research could remain an interesting avenue for future research.

This study could not examine the specific approaches, frameworks, or practices associated with validation of assessment tools and approaches, as the in-depth analysis of validity evidence was not possible with a bibliometric approach. However, the heterogeneity of journals, contexts, assessment tools, authors, and respective training backgrounds reported here could imply heterogeneous approaches to validity and validation. While speculative, it may be reasonable to assume that such a heterogeneous literature, generated by a heterogeneous group of authors, is unlikely to approach validity and validation of assessment in a homogeneous manner [21, 37]. It has been suggested that there is a disconnect between current recommended validation approaches and actual practice [16, 19], and it is possible that this disconnect may be due, at least in part, to different conceptualizations of validity [21]. This study may suggest the presence of multiple conceptualizations of validity and validation; however, this suggestion is based only on the observed heterogeneity of the bibliometric qualities included in this study.

In conclusion, the purpose of this study was to examine the bibliometric characteristics of the literature reporting on validation of assessments within medical education. This literature is growing rapidly, and is quite diverse, which likely suggests that approaches to validation of assessment within medical education are heterogeneous in nature. A gap has been identified between recommended and reported validation practices in medical education assessment literature, and we would agree that better translational work of several relevant assessment frameworks [24, 25, 39] for application in medical and health professions education is one important means for improving the quality and communication of validation studies. There is little evidence to support the unilateral endorsement of one validation framework in the context of assessment in medical education; rather, we would suggest that authors explicitly report which framework was chosen, how it aligns with the purpose, context, and intended use of assessment scores. It is possible that the variation in assessment practices previously documented [15] is reflective of the diversity in assessment tools, approaches, contexts, audiences, constructs, and publication venues represented in this bibliometric analysis of the literature reporting on validation of assessment in medical education.

## Caption Electronic Supplementary Material

ESM-Appendix 1 Search strategy and Appendix 2 Authors with five or more publications included in the study (author in first, last, or sole author position)

ESM-Figure 1 Number of publications reporting validation of assessment across time

ESM-Table 1 Journals with 15 or more published articles included in the study

ESM-Table 2 List of categories representing at least 1% of total publications, and the number of journals associated with each category
