# Supplementary material for: Characterizing the literature on validity and assessment in medical education: a bibliometric study
Source: Perspect Med Educ. 2018 May 23;7(3):182–91. doi: 10.1007/s40037-018-0433-x (PMC6002290; doi:10.1007/s40037-018-0433-x)
Supplement: Supplementary file 1 — ESM-Appendix 1 Search strategy and Appendix 2 Authors with five or more publications included in the study (author in first, last, or sole author position) [file 40037_2018_433_MOESM1_ESM.docx]

**Appendix**

Appendix 1: Medline search strategy:

***Concept 1: Measurement***

1. Educational Measurement/

2. evaluat*.tw.

3. assess*.tw.

4. rating*.tw.

5. rate*.tw.

6. measure*.tw.

7. test*.tw.

8. accura*.tw.

9. perform*.tw.

10. error*.tw.

11. scor*.tw.

12. exam*.tw.

13. (grade* or grading).tw.

14. quiz*.tw.

15. judg*.tw.

16. or/1-15

***Concept 2: Validity***

17. valid*.tw.

18. reliab*.tw.

19. 17 or 18

***Concept 3: Educational Area***

***Undergraduate Medicine Education***

20. Education, Medical, Undergraduate/

21. Education, Medical/

22. Students, Medical/

23. medical student*.tw.

24. clerkship*.tw.

25. (undergraduate* adj3 medic*).tw.

26. or/20-25

***Postgraduate Medicine Education***

27.Education, Medical, Graduate/

28. "Internship and Residency"/

29. residen*.tw.

30. (post?graduate* adj3 medic*).tw.

31. or/27-30

32. 26 or 31

33. 16 and 19 and 32

34. limit 33 to english or french

35. limit 34 to yr=“1999-Current”

Appendix 2. Unique authors with five or more publications combined within sole, first, and last authors included in the database.

| ***Name*** | ***Publications*** |
| --- | --- |
| Van der Vleuten C | 35 |
| Scott DJ | 27 |
| Norman GR | 20 |
| Boulet J | 19 |
| Darzi A | 19 |
| Eva KW | 14 |
| Grantcharov TP | 14 |
| Kerfoot BP | 13 |
| Kreiter CD | 13 |
| Bhatti NI | 12 |
| Hojat M | 12 |
| Dubrowski A | 11 |
| Dunnington G | 11 |
| Durning SJ | 11 |
| Fried GM | 11 |
| McGaghie WC | 11 |
| Regehr G | 11 |
| Ringsted C | 11 |
| Wilkinson TJ | 11 |
| Charlin B | 10 |
| Jones DB | 10 |
| Wayne DB | 10 |
| McLaughlin K | 9 |
| Morgan PJ | 9 |
| O'Sullivan PS | 9 |
| Sarker S | 9 |
| Beckman TJ | 8 |
| Fowler DL | 8 |
| Goff BA | 8 |
| McDougall EM | 8 |
| McManus IC | 8 |
| Schijven MP | 8 |
| Sibert L | 8 |
| Ferguson,KJ | 7 |
| Matsumoto ED | 7 |
| Perkins GD | 7 |
| Vassiliou MC | 7 |
| Donnon T | 7 |
| Iramaneerat C | 7 |
| MacRae H | 7 |
| Naik V | 7 |
| Neary P | 7 |
| Schuwirth LWT | 7 |
| Stefanidis D | 7 |
| Van Zanten M | 7 |
| Vincent C | 7 |
| Wood T | 7 |
| Hulsman RL | 6 |
| Kogan JR | 6 |
| Gallagher AG | 6 |
| Gonnella JS | 6 |
| Hatala R | 6 |
| Holmboe E | 6 |
| Lendvay T | 6 |
| Lurie S | 6 |
| Muijtjens AMM | 6 |
| Pugh CM | 6 |
| Rees JL | 6 |
| Shea JA | 6 |
| Sidhu R | 6 |
| Ten Cate O | 6 |
| Violato C | 6 |
| Wass V | 6 |
| Ziv A | 6 |
| Dagnone JD | 5 |
| Dowell J | 5 |
| Epstein RM | 5 |
| Ferenchick GS | 5 |
| McKinley DW | 5 |
| Nicandri G | 5 |
| Schwartz A | 5 |
| Swanstrom LL | 5 |
| Sweet RM | 5 |
| Yusoff MSB | 5 |
| Brydges R | 5 |
| Buckley CE | 5 |
| Cook DA | 5 |
| De Champlain AF | 5 |
| Downing SM | 5 |
| Driessen EW | 5 |
| Gould JC | 5 |
| Ohdan H | 5 |
| Palter V | 5 |
| Pangaro LN | 5 |
| Reiter HI | 5 |
| Satava R | 5 |
| Smith CD | 5 |
| Weller J | 5 |
| Yudkowsky R | 5 |
