# Supplementary figures and images for: Characterizing the literature on validity and assessment in medical education: a bibliometric study
Source: Perspect Med Educ. 2018 May 23;7(3):182–91. doi: 10.1007/s40037-018-0433-x (PMC6002290; doi:10.1007/s40037-018-0433-x)

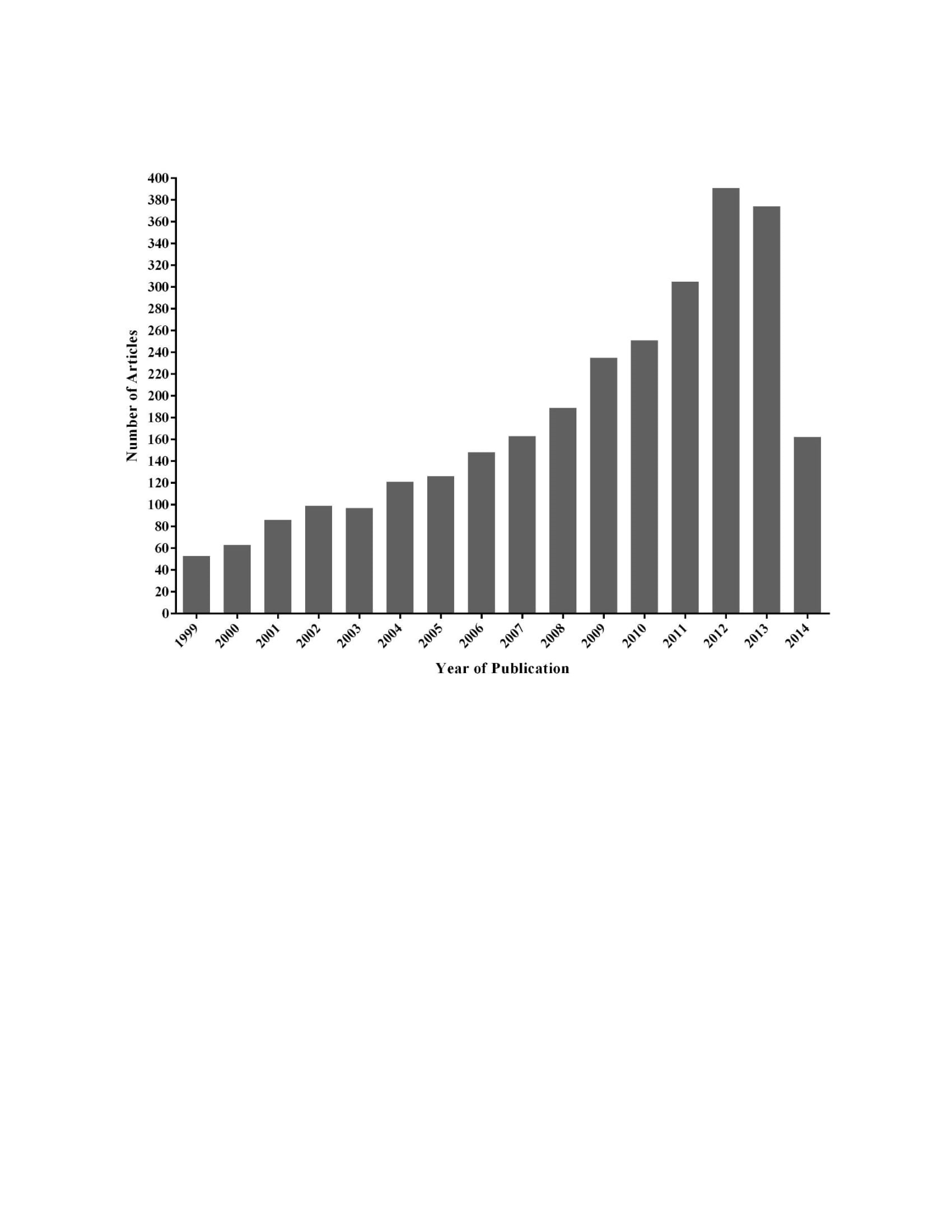

Supplement: Supplementary file 2 — ESM-Figure 1 Number of publications reporting validation of assessment across time [file 40037_2018_433_MOESM2_ESM.jpg]
