# Supplementary material for: Characterizing the literature on validity and assessment in medical education: a bibliometric study
Source: Perspect Med Educ. 2018 May 23;7(3):182–91. doi: 10.1007/s40037-018-0433-x (PMC6002290; doi:10.1007/s40037-018-0433-x)
Supplement: Supplementary file 3 — ESM-Table 1 Journals with 15 or more published articles included in the study [file 40037_2018_433_MOESM3_ESM.docx]

Table 1. Journals with 15 or more published articles included in the study

| ***Journal name*** | ***Number of articles*** | ***%*** |
| --- | --- | --- |
| Medical Education | 211 | 7.4 |
| Academic Medicine | 163 | 5.7 |
| Medical Teacher | 134 | 4.7 |
| Journal of General Internal Medicine | 88 | 3.1 |
| Advances in Health Sciences Education | 85 | 3.0 |
| American Journal of Surgery | 80 | 2.8 |
| BioMed Central Medical Education | 72 | 2.5 |
| Academic Emergency Medicine | 67 | 2.3 |
| Teaching and Learning in Medicine | 61 | 2.1 |
| Surgical Endoscopy | 60 | 2.1 |
| Journal of Surgical Education | 55 | 1.9 |
| Surgical Endoscopy and Other Interventional Techniques | 54 | 1.9 |
| Journal of Urology | 43 | 1.5 |
| Simulation in Healthcare | 33 | 1.2 |
| Patient Education and Counseling | 29 | 1.0 |
| American Journal of Obstetrics and Gynecology | 28 | 1.0 |
| Journal of Surgical Research | 28 | 1.0 |
| Family Medicine | 26 | 0.9 |
| Journal of the American College of Surgeons | 25 | 0.9 |
| Laryngoscope | 25 | 0.9 |
| Academic Psychiatry | 24 | 0.8 |
| Annals of Surgery | 22 | 0.8 |
| Journal of Bone and Joint Surgery | 22 | 0.8 |
| Surgery | 21 | 0.7 |
| Journal of Endourology | 20 | 0.7 |
| Resuscitation | 18 | 0.6 |
| Anesthesiology | 16 | 0.6 |
| Dissertation Abstracts International Section A: Humanities and Social Sciences | 16 | 0.6 |
| Journal of the American Geriatrics Society | 16 | 0.6 |
| Canadian Journal of Emergency Medicine | 15 | 0.5 |
| Otolaryngology - Head and Neck Surgery | 15 | 0.5 |
