# Supplementary material for: Characterizing the literature on validity and assessment in medical education: a bibliometric study
Source: Perspect Med Educ. 2018 May 23;7(3):182–91. doi: 10.1007/s40037-018-0433-x (PMC6002290; doi:10.1007/s40037-018-0433-x)
Supplement: Supplementary file 4 — ESM-Table 2 List of categories representing at least 1% of total publications, and the number of journals associated with each category [file 40037_2018_433_MOESM4_ESM.docx]

Table 2. List of categories representing at least 1% of total publications, and the number of journals associated with each category

| **Category** | **Number of articles** | **% of articles*** | **Number of unique journals** | **% of journals*** |
| --- | --- | --- | --- | --- |
| Education | 769 | 26.9 | 35 | 5.7 |
| General Surgery | 569 | 19.9 | 78 | 12.7 |
| Medicine | 259 | 9.0 | 97 | 15.8 |
| Gastroenterology | 158 | 5.5 | 27 | 4.4 |
| Diagnostic Imaging | 147 | 5.1 | 14 | 2.3 |
| Emergency Medicine | 134 | 4.7 | 17 | 2.8 |
| Gynecology | 107 | 3.7 | 18 | 2.9 |
| Internal Medicine | 104 | 3.6 | 7 | 1.1 |
| Public Health | 103 | 3.6 | 13 | 2.1 |
| Urology | 103 | 3.6 | 17 | 2.8 |
| Medical Informatics | 90 | 3.1 | 16 | 2.6 |
| Obstetrics | 88 | 3.1 | 12 | 2.0 |
| Health Services Research | 78 | 2.7 | 17 | 2.8 |
| Anesthesiology | 77 | 2.7 | 13 | 2.1 |
| Pediatrics | 69 | 2.4 | 29 | 4.7 |
| Otolaryngology | 63 | 2.2 | 17 | 2.8 |
| Primary Health Care | 61 | 2.1 | 19 | 3.1 |
| Psychiatry | 50 | 1.7 | 23 | 3.7 |
| Health Services | 46 | 1.6 | 19 | 3.1 |
| Ophthalmology | 40 | 1.4 | 19 | 3.1 |
| Orthopedics | 40 | 1.4 | 12 | 2.0 |
| Nursing | 37 | 1.3 | 8 | 1.3 |
| Radiology | 37 | 1.3 | 15 | 2.4 |
| Pulmonary Medicine | 34 | 1.2 | 12 | 2.0 |
| Neurology | 32 | 1.1 | 14 | 2.3 |
| Vascular Diseases | 30 | 1.0 | 11 | 1.8 |

******* *Discipline categories’ representations in total articles and unique journals may sum to more than 100% as each article and unique journal could contain more than one discipline category.*
